# Evaluation of hydrophobic‐interaction chromatography resins for purification of antibody‐drug conjugates using a mimetic model with adjustable hydrophobicity

**DOI:** 10.1002/jssc.201900895

**Published:** 2020-04-08

**Authors:** Egbert Müller, Manuela Sevilla, Patrick Endres

**Affiliations:** ^1^ Tosoh Bioscience GmbH Im Leuschnerpark 4 Griesheim 64347 Germany

**Keywords:** antibody drug conjugates, drug antibody ratio, fluorescein 5‐isocyanate, hydrophobic interaction chromatography, purification

## Abstract

Antibody drug conjugates are cytotoxic pharmaceuticals, designed to destroy malignant cells. A cytotoxic molecule is attached to an antibody that binds specific to a cancer‐cell surface. Given the high toxicity of the drugs, strict safety standards have to be kept. For this reason, an antibody drug conjugates model was developed with fluorescein 5‐isothiocyanate as the nontoxic payload surrogate. Due to the similar hydrophobicity, this model is used to establish a suitable purification process and characterization method for antibody drug conjugates. Because of the pH dependent solubility of fluorescein, the hydrophobicity of conjugates can be modulated by the pH value. Based on the complex heterogeneity and hydrophobicity of the conjugates a chromatographic purification is challenging. Hydrophobic interaction chromatography is used for analytical as well as for preparative separation. Because of the increased hydrophobicity of the conjugates compared to native antibody, hydrophobic interaction chromatography often suffer from resolution and recovery problems. Conjugates were separated differing on the number of payloads attached to the antibody. For this matter, the drug–antibody ratio is determined and used as a quantitative term. The conjugates are purified at high recoveries and resolution by step gradients using suitable resins, allowing the separation of the target drug–antibody ratio.

**Article Related Abbreviations**
ADC: antibody–drug conjugate
CV: column volume
DAR: drug–antibody ratio
FITC: fluorescein 5‐isocyanate
HIC: hydrophobic interaction chromatography

## 1 INTRODUCTION

The method of a cancer treatment depends on the tumor location and the stage of the disease. Above all, the main goal is to remove malignant cells completely with a minimal damage of the rest of the body.

Antibody–drug conjugates (ADC) offer the possibility to reach this goal; attaching cytotoxic molecules to a monoclonal antibody (mAb) through a linker. The antibody recognizes specifically the antigen that is amply overexpressed on the surface of the cancer cell. The cytotoxic drug is then delivered selectively killing the target cell. Due to this specific targeting, an ADC has lower side effects and a wider therapeutic window, compared to other chemotherapeutic agents [1]. Currently, seven ADCs are approved, but over 60 are being generated in clinical trials [2]. The ADC development consists of several steps such as: mAb activation, conjugation, purification, formulation, and storage. The fabrication of this therapeutics is very challenging. There are several methods to conjugate ADCs; enzymatic or chemical conjugation, and through a cleavable or a noncleavable linker. The differences between each method can be found in the literature [3, 4]. The most popular way to do it has been to conjugate the antibody and payload through the antibody's amino acids such as Lysine or Cysteine. This method leads to a nonsite specific‐conjugation that leads in turn to a heterogeneous distribution. The number of toxins attached to an antibody can vary between zero and several drugs per antibody, and is characterized by the drug–antibody ratio (DAR). Tassi et al. reviewed the importance to determine the average DAR, considering the delivery and distribution of the therapeutic into the target cells [5].

Furthermore the purification process is also complex due to the heterogeneity of the conjugates. The main challenges are the removal of unconjugated antibody if desired and the remaining free drug, as well as the separation of conjugates with different DARs. The DAR is the key value for the production process and it correlates with the potency of the mAb for damage of malignant cells. The high DARs are associated with high toxicity for both healthy tissue and malignant cells, and it can also cause aggregation, affecting the stability of the ADC [6]. On the other hand, a low DARs could affect the potency of the therapeutics reducing the antitumor efficacy [4, 7].

The purification process of ADC has to be carefully designed regarding safety measures, and it is the reason why the ADC‐surrogates are consequently a good option for optimization of the purification process. They can serve as model with similar physicochemical properties but containing a nontoxic hydrophobic molecule with a similar structure as the toxic‐payload.

In this work, a purification process has been developed for a mimetic ADC‐model. Fluorescein 5‐isothiocyanate (FITC) has been selected as the nontoxic payload. The chemical structure of FITC is composed of aromatic rings, which makes it similar to the hydrophobic drugs. Previous studies have already shown a fluorescein‐labeled monoclonal antibody as a model‐ADC [8]. FITC is conjugated to the mAb through Lysine, this method leads to a nonsite specific‐conjugation that turns to a heterogeneous distribution.

Chromatography can be used during drug discovery, characterization, and analysis of various therapeutics [9]. Several methods are known in the literature to analyze and characterize ADCs [10]. Reversed‐phase high‐throughput chromatography, which separates according to differences in hydrophobicity of the analytes, is widely applied to determine the DAR distribution. The selectivity can be improved by modifying the base material [11], changing the pH‐value [12] or by implementing an alternative mobile phase modifier [13]. However, this method usually requires the addition of organic modifier, which alters the structure and therefore the efficacy of the ADC, in this matter is not always suitable for the characterization [10] and for the purification. ADCs demand monitoring multiple classes of analytes, therefore a multipath LC–MS detection is suitable for the characterization of these therapeutics [14]. Another method known to characterize ADCs is ion‐mobility spectrometry, this approach offers the detection of all conformation species of ADC, including the average DAR [15].

Hydrophobic interaction chromatography (HIC) is often used for the characterization of ADCs [16]. The main advantage of HIC is the performance under non‐denaturing conditions, which offers the isolation of chromatographically pure species permitting their further analysis. The quantification of the payload‐to‐antibody ratio can therefore determine with HIC since the cytotoxic drugs are hydrophobic and alter the physicochemical properties of the antibody [17].

In this work, several HIC resins with different ligands and hydrophobicity level were tested to establish a purification method. Various HIC resins have been compared in order to find the best recovery and selectivity of the conjugates. An estimation of the DAR could be calculated by determining the absorbance of the conjugate at 280 nm for the mAb and at 495 nm for FITC, this has been used as a quantitative term to characterize the conjugates. Using this strategy, the most important challenges in optimization the separation of heterogeneously loaded hydrophobic ADCs have been overcome, the non‐conjugated payload has been isolated and the target conjugate with optimal DAR could be purified.

## 2 MATERIALS AND METHODS

### 2.1 Monoclonal antibody

The monoclonal antibody was purchased from Bioceros (Utrecht, the Netherlands). The mAb was produced in Chinese hamster ovary cells. Prior to conjugation, the mAb was purified in a 5 mL prepacked MiniChrom AF‐rProtein A HC‐650F column (Tosoh Bioscience, Griesheim, Germany).

### 2.2 Antibody–payload conjugation

For the preparation of the antibody‐FITC conjugates, circa. 20 mL of the purified antibody was diafiltrated to a concentration of 5.0 mg/mL in sodium carbonate buffer pH 9.0 and incubated with FITC (Merck, Darmstadt, Germany) in a thermomixer comfort (Eppendorf, Hamburg, Germany) for 8 h at 4°C. FITC was dissolved in *N*,*N*‐Dimethylformamide (Merck) to a final concentration of 1.0 mg/mL and was added in 10 × 10 μL steps to a reaction vessel with 950 μL mAb solution. This equals a ratio of 8 mol FITC to 1 mol mAb. The samples were mixed at 500 rpm and covered to avoid light contact. After the conjugation, the unconjugated FITC was removed of the sample. For that manner, the sample was dialyzed in 0.1 mol/L sodium phosphate buffer pH 7.0 with a Spectra/Por 7 Dialysis 3.5 kDa membrane (Spectrum Laboratories, Rancho Dominguez, CA, USA) for 12 h.

### 2.3 Estimation octanol/water partition coefficient of fluorescein 5‐isothiocyanate

The octanol/water partition coefficient for FITC was measured at different pH values. Fluorescein sodium salt (Merck) was used due to the poor solubility of FITC in water. Various buffers with a pH range between 5.0 and 9.0 were prepared and mixed with 1‐octanol (Merck). The Fluorescein salt was added to a final concentration of 1.0 mg/mL. After 1 h of mixing at room temperature, a sample of the aqueous phase and one of the octanol phase were measured at 460 nm in an optical reader. The concentration in each phase was determined through a calibration curve at each pH value with the UV absorbance of FITC‐salt. Then, Equation (1) was used to calculate the partition coefficient.

(1) $$\log P_{\mathrm{ow}}=\;log\frac{c_{i,\mathrm{octanol}}}{c_{i,\;\mathrm{water}}}$$

### 2.4 Analytical chromatography

The antibody and the conjugates were analyzed by analytical size exclusion chromatography (SEC) and HIC. For the analytical SEC a TSKgel G3000SW_XL_ 7.8 mm ID × 30 cm L (Tosoh Bioscience) was used with 0.1 mol/L PBS‐Buffer, pH 6.7, at 1.0 mL/min. A TSKgel Butyl‐NPR 4.6 mm ID × 10 cm L column (Tosoh Bioscience), with 2.5 μm non‐porous polymethacrylate particles, was selected for the analytical HIC. Note that 0.1 mol/L sodium phosphate buffer, pH 7.0 containing 2.0 mol/L ammonium sulfate was used during load and during elution was used the same buffer without addition of ammonium sulfate. All experiments were performed in a Dionex Ultimate 3000 HPLC system (Thermo Fischer Scientific, Dreieich, Germany).

### 2.5 Preparative chromatography

Various HIC‐Resins from Tosoh Bioscience GmbH were tested in terms of resolution and recovery at different pH values. The HIC resins listed in Table 1 were packed in Omnifit Benchmark glass columns (Diba Industries, Cambridge, UK) 6.6 mm ID × 10 cm L (V = 3.42 mL). Only columns with a peak symmetry between 0.8 and 1.4 were used for further experiments. An ÄKTA Avant system (GE Healthcare, Chicago, IL, USA) was used for the experiments. The buffer selection for the method was in dependence of the pH, to test the resolution the conjugate in the different HIC‐resins, a pH value of 6.5 was selected and sodium phosphate buffer was used. The recovery was determined between pH 5.0 and 9.0, where different buffers were selected in dependence on their buffer properties. Sodium acetate was used for the pH adjustment in the region between 5.0 and 5.5. For pH values between 6.0 and 8.0, sodium phosphate buffer was used and Tris‐HCl was used for pH 9.0. The binding buffer contained 1.5 mol/L ammonium sulfate, which was selected as the optimal salt, resulting in maximal concentration for the mAb and the conjugates in previous investigations (data not shown). For the elution buffer, the pH corresponding buffer with no salt added has been used.

**TABLE 1:** Specifications of selected HIC gels

Resin	Particle size (μm)	Pore size (nm)
TOYOPEARL Butyl‐650S	35	100
TOYOPEARL Phenyl‐650S	35	100
TOYOPEARL Phenyl‐650 M	65	100
TOYOPEARL Phenyl‐600 M	65	75
TSKgel Phenyl‐5PW (20)	20	100
TOYOPEARL PPG‐600 M	65	75

The samples were adjusted to a final concentration of 1.5 mg/mL with the correspondent buffer, the concentration was estimated with a spectrophotometer NanoDrop 2000c (Thermo Fisher Scientific, Dreieich, Germany) at 280 nm. For the method, the columns were equilibrated for five column volumes (CV), then 1.0 mL sample were injected into the column, with the same equilibration buffer a wash step was set for two CV. For the elution, the salt concentration was decreased gradually during a linear gradient over 60 min. After each method the columns were clean with sodium hydroxide. A linear flow velocity of 150 cm/h (0.85 mL/min) was used during the method. The recovery of the ADC surrogates was calculated with equation (2).

(2) $$\mathrm{Recovery}\;\%\;=\frac{A_{\mathrm{Elution}}}{A_{\mathrm{Feed}}}\;*\frac{V_{\mathrm{Elution}}}{V_{\mathrm{Feed}}}*100$$

## 3 RESULTS & DISCUSSION

### 3.1 Fluorescein 5‐isothiocyanate dependency on the pH value

**FIGURE 1:**
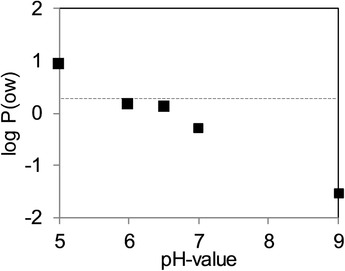
Octanol–water partition coefficient for fluorescein sodium salt in dependence on the pH value. The fluorescein salt was mixed at 1.0 mg/mL in the two immiscible phases. The concentration in each phase was calculated with a calibration curve through the UV absorbance

One of the challenges for the development of the mimetic ADC is to ensure that the characteristics of the model are comparable with the real drugs. The hydrophobicity is one the most important attributes. In the case of the mAb–FITC conjugates, the hydrophobicity of FITC is highly dependent of the pH value. This relationship has been investigated by determining the octanol/water partition coefficient of FITC. As shown in Figure 1, the $\log P_{\mathrm{OW}}$ presents a big influence by changing the pH value. At both pH 5.0 and 5.5, the logarithmic value of the partition coefficient $(\log P_{\mathrm{OW}})\;$reaches a value of 1.0, meaning that the substance is hydrophobic. The limit between hydrophobic and hydrophilic behavior of FITC is found between pH 6.5 and 7.0. At pH 7.0 and higher, FITC behaves completely hydrophilic. These results can be compared with the investigation of Oba and Poulson, where the $\log P_{\mathrm{OW}}$ for FITC is indicated as 0.8 at a pH value of 6.5. And at a higher pH value, FITC does not longer present a hydrophobic character [18]. Toxic payloads use for real ADCs present higher $\log P_{\mathrm{OW}}$ values, Maytansine presents a coefficient of 1.99 [19], Dolastitin 10 is indicated to have a $\log P_{\mathrm{OW}}$ of 3.4 and MMAE presents a value of 2.2 [20]. Although FITC present a lower partition coefficient as the real payloads, a pH value of 6.5 was chosen as appropriate for the HIC process with the ADC surrogates. FITC presents already a hydrophobic character at this value and the stability of the mAb and conjugates is not compromised. This is in agreement with the investigation of Rodriguez‐Aller et al. with a real ADC, when the pH of the mobile phase was adjusted to the value between 6.4 and 7 to be most appropriate for the separation [21].

### 3.2 Purification process

The conjugation and purification of ADC require conditions that do not affect the molecular structure of any components involved [4]. One of the many challenges of the purification process of ADC is the removal of the unconjugated toxin. Wakankar et al. describe the free drug molecules as a differential in toxicity and a cause for potential safety issues [10]. Therefore, it is important to detect and remove the unconjugated payloads to ensure the stability and safety of the therapeutic.

**FIGURE 2:**
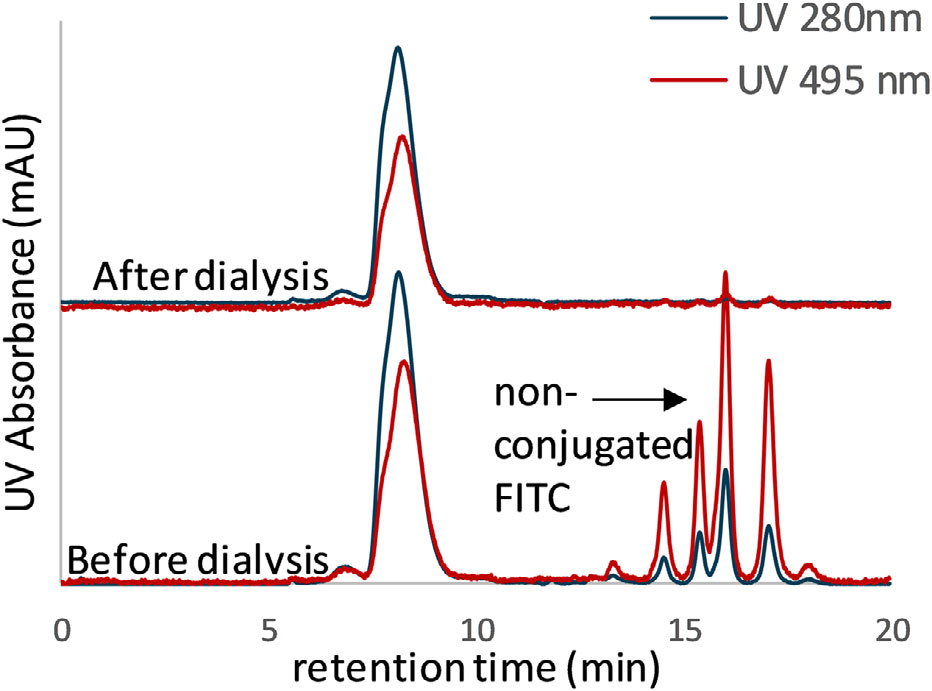
ADC surrogate analyzed in a TSKgel G3000SWxl (7.6 mm ID × 30 cm L). The sample was tested before and after performing the membrane dialyzed to remove the non‐conjugated FITC

**FIGURE 3:**
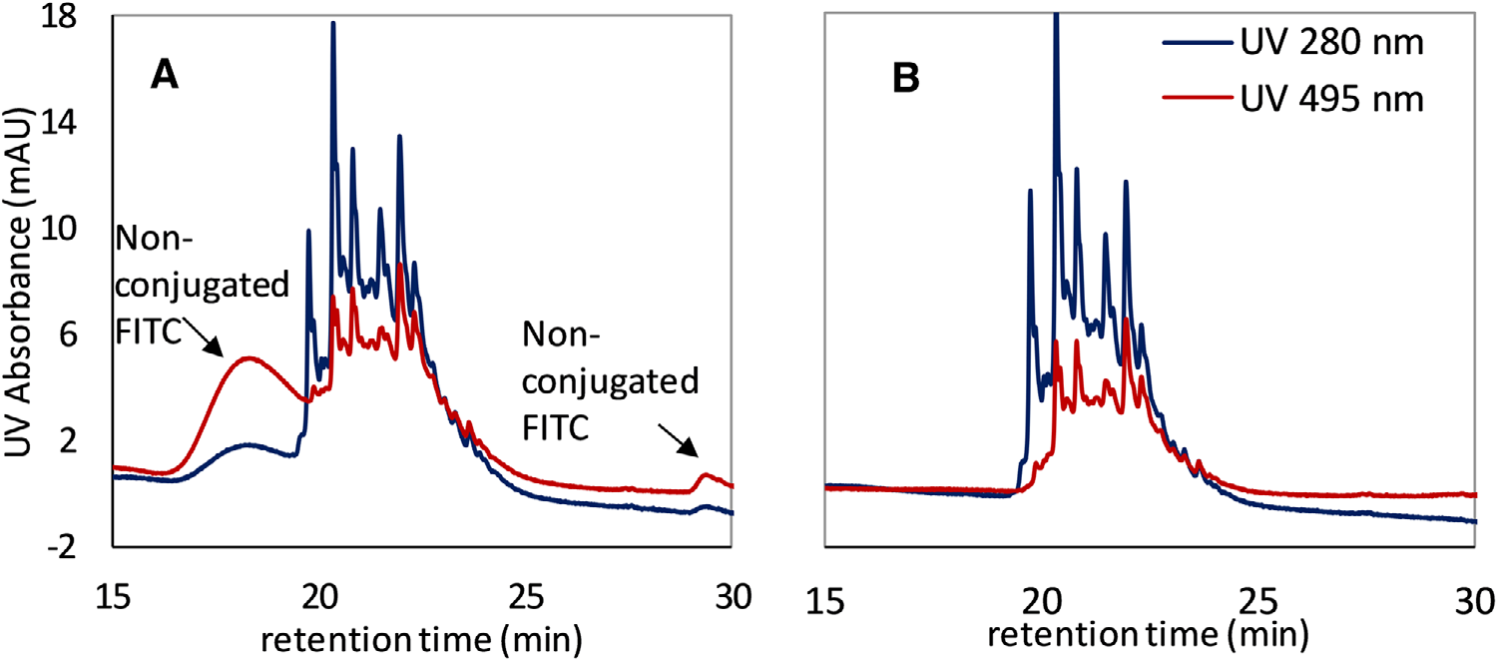
ADC‐Surrogates analyzed in a TSKgel Butyl‐NPR column (4.6 mm ID × 10 cm L) in terms of stability. Non‐conjugated FITC elutes before and after the conjugates (A). After performing the dialysis membrane, free FITC is completely removed and any aggregation is detected (B)

After the conjugation of the mimetic ADC, a SEC analysis has been performed (Figure 2), where non‐conjugated FITC is detected. For this reason, after the coupling reaction, a membrane dialysis has been performed in order to remove the non‐conjugated payload. Schwarz et al. reported that the attachment of hydrophobic payload to form an ADC enhances hydrophobicity‐driven aggregation. The aggregation may indicate limited stability for the ADC that may results in the toxicity effects of the drug [6]. To ensure the complete removal of free FITC and the stability of the conjugates during this step, the sample has been tested with an analytical HIC and SEC. Figures 2 and 3 show the chromatogram of the conjugates before and after the dialysis, where the stability of the conjugates can be confirmed. There is no detection of free FITC after this step, and no aggregates or shifting of the retention time.

The analytical HIC illustrated in Figure 3 shows that the free FITC elutes partly before and after the ADC‐surrogates. This could be caused by the differences in hydrophobicity of the free molecules, probably due to aggregation of the FITC‐molecules.

### 3.3 Recovery in dependency of the pH value

**FIGURE 4:**
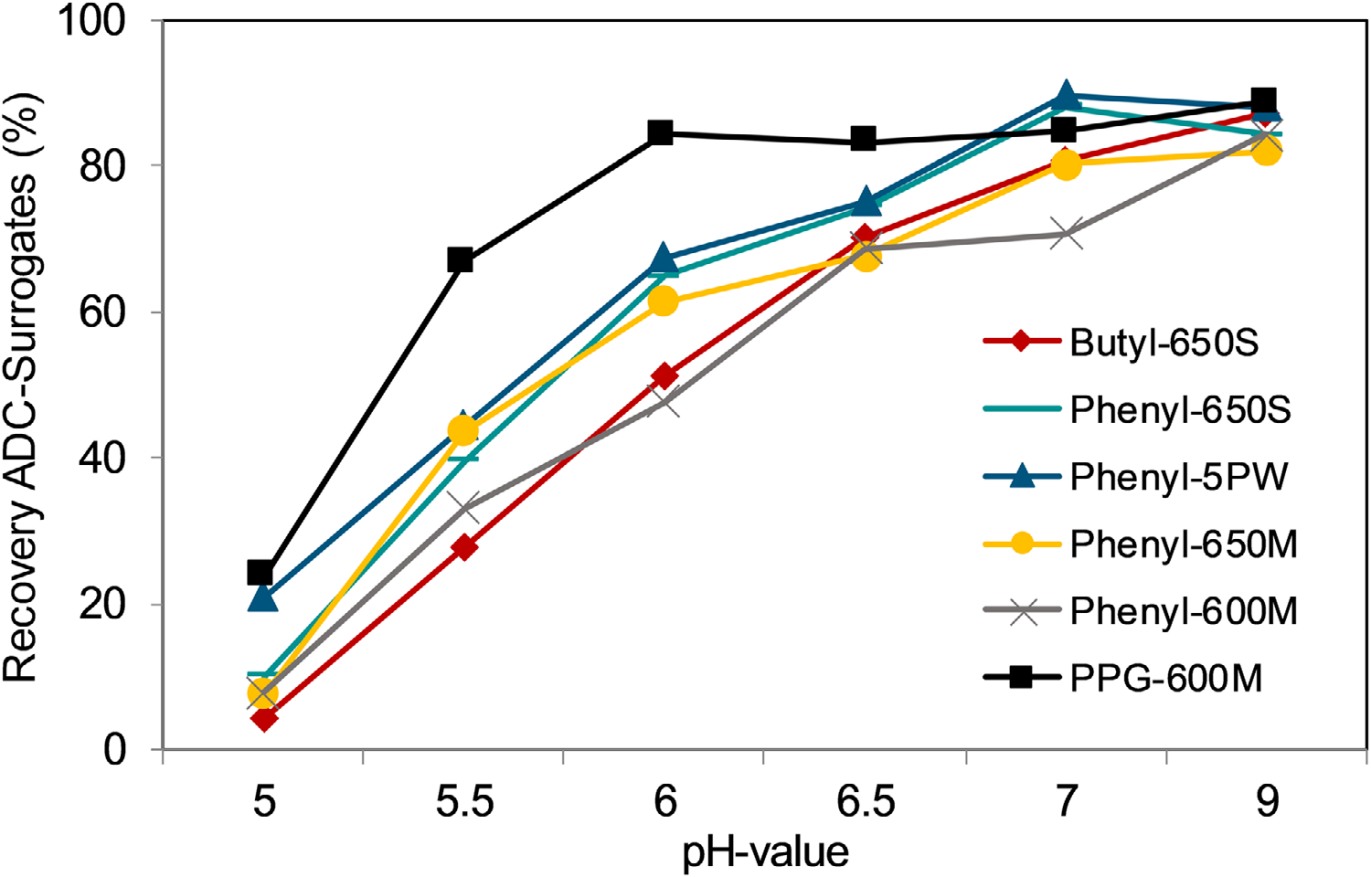
The recovery of mimetic molecules was determined in dependency of the pH‐value. The HIC‐Resins were packed in glass columns (6.6 mm id × 10 cm L). At lower pH values, the conjugates present a high hydrophobicity hence to a low recovery

To determinate a suitable resin for the purification process, the recovery of the ADC surrogates has been determined for separation efficiency of HIC gels at various pH values. Fausnaugh et al. described the protein surface hydrophobicity as the most affecting factor between the ligand of the resin and the retention of the protein [22]. In this work, the recovery of the mimetic molecules is mainly affected by the pH‐dependent hydrophobicity of FITC (Figure 4). At a low pH, the surrogates are most hydrophobic, hence the binding strength in the HIC columns is higher and results in a low recovery. On the other hand, at pH values of 7.0 and 9.0, the ADC surrogates elute almost completely from all resins, because the hydrophobicity decreases. The recovery is also influenced by the ligand hydrophobicity. Butyl is considered to be purely hydrophobic, whereas PPG (polypropylene glycol) presents a more polar‐hydrophobic ligand. Comparing the recovery at low pH, PPG‐600 M reaches the highest recovery of all gels. Already at pH 5.5 a recovery of 70% is achieved due to the relatively hydrophilic ligand together with a high binding capacity. On the other side, the resin with butyl as hydrophobic ligand reaches a high recovery at pH 6.5 and increases drastically at pH 7.0 when the surrogates are already hydrophilic. The property of the ADC surrogates to adjust their hydrophobicity with the pH value can be used to tune their hydrophobicity in order to mimic different behavior of real ADCs. With this novel strategy, one model molecule can mimic different molecules and a more precise purification process with HIC can be developed depending on the hydrophobicity of the desired ADC.

### 3.4 Resolution and drug–antibody ratio estimation

The method development of an ADC‐purification process is not straightforward [23]. Several parameters have to be taken in consideration to achieve an optimal HIC method for mAbs and ADCs. It is necessary to optimize the pH, the salt type and amount in the equilibration buffer, and the gradient.

**FIGURE 5:**
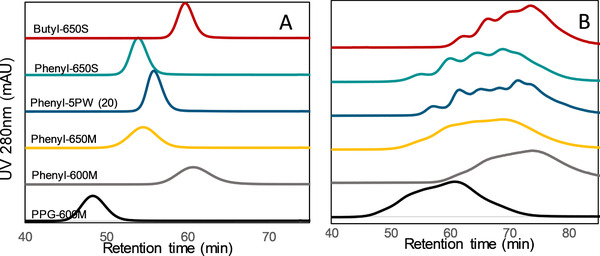
Comparison of resins with different HIC‐ligands in a linear gradient from 0 to 100% mobile phase B. The mobile phase A was 0.1 mol/L sodium phosphate buffer with 1.5 mol/L ammonium sulfate salt with a pH value of 6.5. The mobile phase B was the same buffer without salt. The influence of the different hydrophobic ligands is reflected in the shift of the retention time of the mAb‐elution (A). The HIC‐Resins with smaller particles present a better selectivity between the conjugates (B)

The main goal in the purification process is to isolate the different DARs of the conjugates. A good resolution and selectivity of the resin for the separation of conjugates is needed, therefore different chromatographic gels with different characteristics have been tested to select the most appropriate one to reach this goal, such as different ligand hydrophobicity, small and medium particle size, and various pore size. At first pure mAb was injected in the HIC columns, Figure 5A illustrates the absorbance at 280 nm in dependence of the retention time for each HIC gel. As expected, there are differences on the elution time of the sample due to the different hydrophobicity of each gel. Each mAb present different hydrophobicity that can cause a shift on the retention time as shown in the literature [24], that is why a method development is necessary for each antibody and ADC.

The mAb elutes earlier from PPG‐600 M, given that the ligand is the less hydrophobic than phenyl and butyl. The resins with different pore size show also differences on the retention time. Phenyl‐650 M and Phenyl‐600 M, with a pore size of 100 and 75 nm, respectively, evidence a shift in the retention time at smaller pore size. This can be presumable explained by the slower diffusion of the molecules on the smaller pores, or larger surface area and subsequent higher hydrophobicity on the resin smaller pore size.

The same experiment was performed with the ADC surrogates to investigate the selectivity of the DARs in the different HIC resins (Figure 5B). The relationship between the different ligands, and pore size of the resins and the retention time show the same sequence for the ADC as for the mAb.

In terms of separation and resolution of the different conjugates of the ADC surrogates, the particle size shows a great influence. A separation of the ADC surrogates into the peaks or conjugates was only possible with resins with a particle size of 35 μm or less, like Butyl‐650S, Phenyl‐650S, and Phenyl‐5PW.

Single ADC surrogates are composed of various DARs. In order to ensure both highest efficiency and lowest toxicity of the drug, there may be a desired to remove high and low DAR species. The unconjugated protein or low DARs will cause lower efficacy of the drug, whereas the conjugates with high DARs are expected to aggregate and be more toxic, which can cause severe side effects [25, 26]. The decreased stability is caused by the fact that fewer disulfide bonds are available to hold the quaternary structure together [4, 27]. Additionally the ADCs with higher DAR undergo a rapid clearance within the human organism [28].

**FIGURE 6:**
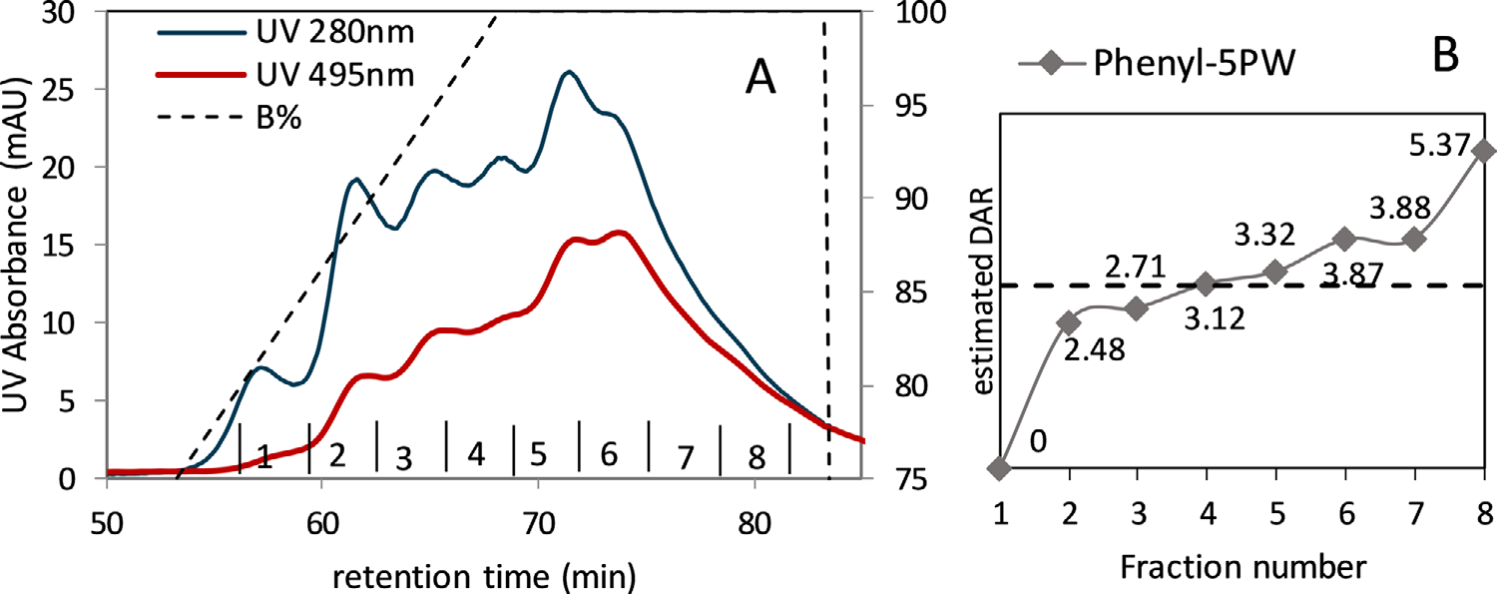
The best selectivity of the conjugates was reached with the TSKgel Phenyl‐5PW (20) at a linear gradient from 0 to 100% no salted B Buffer. The fractions one to eight were collected and analyzed to confirm the influence of the hydrophobicity in each peak (A). The estimated DAR increases with the elution time of each fraction (B)

HIC is the method of choice to determine the drug‐load distribution for ADCs with hydrophobic payloads as a consequence of the increasing hydrophobicity [29]. According to Fekete et al., the reason for this phenomena is that the payloads are hydrophobic, conjugation increases the hydrophobicity of the protein, and therefore, retention increases with the conjugation number [30]. To confirm this relation in the case of the ADC surrogates, the different fractions of the preparative HIC were collected and analyzed in the analytical Butyl‐NPR column. For preparative chromatography, TOYOPEARL Phenyl‐5PW was chosen because of its good selectivity (see Figure 6A). The absorbance at 495 nm increases with the elution time. The fractions corresponding to each peak were collected and analyzed in the TSKgel Butyl‐NPR.

The drug–antibody ratio is one of the most important critical quality attribute of ADCs. It describes the number of payloads attached to an antibody, therefore the number of payloads that can be delivered by a single antibody to the cancer cell. In this work, the DAR was estimated through the UV absorbance at 280 nm for the mAb and at 495 nm for the FITC and calculated with the below equation.

(3) $$DAR\;=\;\frac{2.77*\;A_{495}}{A_{280}-\left(0.35*A_{495}\right)}$$

where the value 0.35 is the correction factor due to the absorbance of FITC at 280 nm [31]. It was calculated that in average a mAb is conjugated with three FITC‐molecules.

Consequently, this value can be compared with the ADC‐model presented in Arakawa's work, which reported a labeling ratio of 2.8 mol FITC per mol protein [8]. In the case of heterogeneous hydrophobic ADCs through lysine conjugation, the literature reports that the average DAR ranges from 3 to 4 [32], meaning that the average DAR of the ADC surrogates is comparable with real ADCs.

To verify the FITC/mAb ratio, an estimated DAR was determined with the absorbance in each fraction. The trend curve of the DARs in dependence of the retention time of each fraction is shown in Figure 6B. The DAR values increase along the retention time from 0 to a value of 5.3. This result confirms that the degree of hydrophobicity is proportional to the DAR. It can be stated that HIC can be used not only for the characterization of ADC but also the purification and separation of the different DARs.

### 3.5 Optimization of the elution gradient

The conjugates in this work are divided in three different DAR groups. The first one has an estimated highest DAR of 5–6, the second one is the group with a low DAR of 0–1. Finally, the majority represents the third group with the average DAR of 3. As cited before, the real challenge in heterogeneous ADCs is to separate the unconjugated drug, the both groups with low DAR and high DAR from the most appropriate group with a DAR between 3 and 4. It was shown that TOYOPEARL PPG‐600 M is the most appropriate resin in terms of recovery for the hydrophobic molecules. Unfortunately, it does not present sufficient selectivity and resolution of the DARs, and it does not ensure a sufficient enrichment of the group with an optimal DAR value. To improve the separation of the conjugates, a step gradient was performed to optimize the purification method. Cusumano et al., who developed a separation method in HIC for mAbs and ADCs, conclude that the only relevant parameter for improvement selectivity for the separation of DAR species is the gradient steepness [33].

**FIGURE 7:**
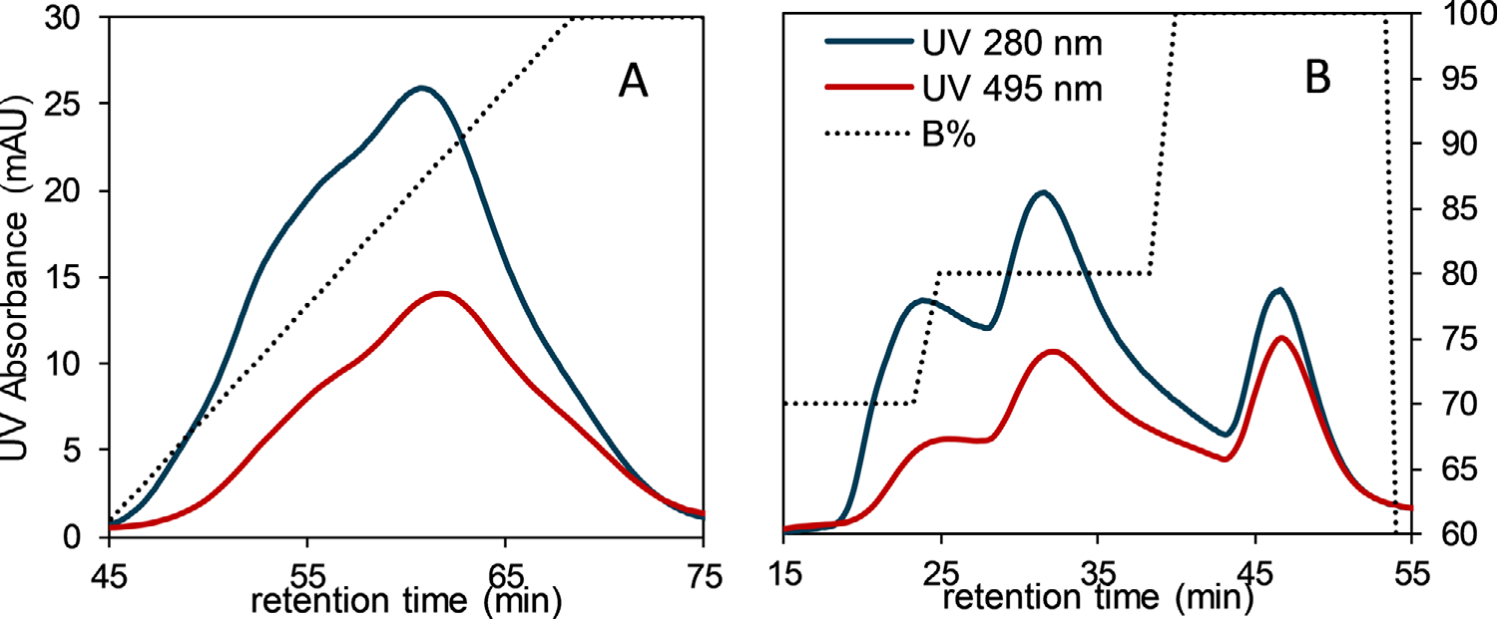
ADC surrogates analyzed in TOYOPEARL PPG‐600 M, which presented the best recovery of the HIC‐Resins. A linear gradient was performed but no selectivity of the conjugates was appeared (A). For a process optimization a step gradient with 70%, 80% and 100% was run for 5 CV each. The ADC surrogates could be separated in three DAR groups (B)

The result of the HIC performed with a linear gradient, shown in Figure 7A, was taken as reference to apply the steps. The gradient was adjusted with decreasing concentration of salt buffer, and it was kept constant in each step for 5 CV. In Figure 7A the ADC‐surrogates elute as a single peak and no resolution can be observed. Based on this result, the step gradient was set from 70% buffer with low salt concentration (Buffer B) to 90% Buffer B, and finally to 100% B. Corresponding chromatogram is shown in Figure 7B. The ADC surrogates elute in three different peaks, one in each step. The percentages of the different DAR groups were calculated from each peak area. The first peak or the low DAR represent a total of 26.7% of the total area, 51.5% represents the intermediate DAR, and 21.8% the highest DAR. In order to confirm the DAR value, the estimated DAR of these three peaks was calculated according to Equation (2). The first peak presents a DAR value of 1.05, the second represents a DAR of 2.7, and the third has a value of 4.86. In this manner, it can be proven that each peak represents each group of DAR values, as mentioned above. A step gradient constitutes an improvement in selectivity in HIC columns with bigger particle sizes. The average DAR represents the main part eluted, which demonstrates that this method is suitable to separate targeted DAR‐group.

## 4 CONCLUSION

A purification process was developed for the separation of ADC surrogates that overcomes the ADC purification challenges. The separation of the different conjugates has been accomplished with HIC, due to an increase on the hydrophobicity with the number of payloads attached. After the removal of the non‐conjugated payload, the surrogates have been analyzed on an HIC‐column in terms of stability and quantification of drug load species. To characterize the conjugates, the drug–antibody ratio has been estimated, the mimetic ADC presents an average DAR of 3.0, which is comparable with real ADC. The recovery and resolution of the conjugates has been investigated by use of different HIC resins. The best recovery has been achieved with a more polar resin. However, the resolution of the different DARs was insufficient. A good resolution of the conjugates has been achieved by use of resins with a particle size of 35 μm or smaller using a linear gradient. In terms to reach the best recovery possible, a step gradient has been developed for resins with bigger particle size, resulting in the separation of the conjugates in three groups. A group of inefficient ADCs with a low DAR, the second group of toxic ADCs with a high DAR, and finally a target group of efficient ADCs with a calculated DAR value of 2.7. The pH dependency of FITC on the hydrophobicity enable the development of the purification process of various ADC models. With variable pH value, the hydrophobicity of the molecules can be adjusted and the best parameters and resin can be selected for each hydrophobicity level.

This publication showed that HIC can be applicable for the purification of the different DAR species of heterogeneous hydrophobic ADCs and not only for the characterization.

Based on this investigation, we recommend a more polar HIC resin at the time to purify high hydrophobic ADC. A method development is necessary, a step gradient is optimal for the separation in the different species, and a resin with smaller particle sizes offers a better resolution.

## CONFLICT OF INTEREST

The authors have declared no conflict of interest.
